# Success of tumorsphere isolation from WHO grade IV gliomas does not correlate with the weight of fresh tumor specimens: an immunohistochemical characterization of tumorsphere differentiation

**DOI:** 10.1186/s12935-016-0350-1

**Published:** 2016-09-27

**Authors:** Kyoung Su Sung, Jin-Kyoung Shim, Ji-Hyun Lee, Se Hoon Kim, Sohee Park, Tae-Hoon Roh, Ju Hyung Moon, Eui-Hyun Kim, Sun Ho Kim, Su Jae Lee, Yong Min Huh, Seok-Gu Kang, Jong Hee Chang

**Affiliations:** 1 Department of Neurosurgery, Brain Tumor Center, Severance Hospital, Yonsei University College of Medicine, 50-1 Yonsei-ro, Seodaemun-gu, Seoul, 120-752 Republic of Korea; 2 Department of Pathology, Brain Tumor Center, Severance Hospital, Yonsei University College of Medicine, 50-1 Yonsei-ro, Seodaemun-gu, Seoul, 120-752 Republic of Korea; 3 Department of Biostatistics, Graduate School of Public Health, Yonsei University, 50-1 Yonsei-ro, Seodaemun-gu, Seoul, 120-752 Republic of Korea; 4 Department of Life Science, Research Institute for Natural Sciences, Hanyang University, 222 Wangsimni-ro, Seongdong-gu, Seoul, 133-791 Republic of Korea; 5 Department of Radiology, Brain Tumor Center, Severance Hospital, Yonsei University College of Medicine, 50-1 Yonsei-ro, Seodaemun-gu, Seoul, 120-752 Republic of Korea

**Keywords:** WHO grade IV glioma, Isolation, Tumorsphere, Weight, Fresh specimen

## Abstract

**Background:**

A trend of stage-by-stage increase in tumorsphere (TS) formation from glioma samples has been reported. Despite this trend, not all surgical specimens give rise to TSs, even World Health Organization (WHO) grade IV gliomas. Furthermore, it has been reported that differences in overall survival of primary glioblastoma patients depends on the propensity of their tumors to form TSs. However, the weights of fresh specimens vary from one surgical isolate to the next.

**Methods:**

Accordingly, we evaluated the relationship between the weights of surgical specimens in WHO grade IV gliomas with the capacity to isolate TSs. Thirty-five fresh WHO grade IV glioma specimens were separated into two groups, based on whether they were positive or negative for TS isolation, and the relationship between TS isolation and weight of surgical specimens was assessed.

**Results:**

We observed no significant difference in the weights of surgical samples in the two groups, and found that the optimal weight of specimens for TSs isolation was 500 mg.

**Conclusion:**

Thus, contrary to our expectations, the ability to isolate TSs from WHO grade IV glioma specimens was not related to the weight of fresh specimens.

## Background

A subpopulation of glioblastoma (GBM) tumor cells possesses the ability to undergo neural differentiation and induce tumorigenesis [1–3]. When cultured under appropriate conditions in vitro, this population of tumor cells gives rise to gliomaspheres, referred to more generically as tumorspheres (TSs). TSs have been isolated from various malignant tumors, including breast [4], prostate [5], bone [6], colon [7], kidney [8] and lung [9], as well as brain [3, 10–14]. It was previously reported that the rate of isolation of TSs increase as the World Health Organization (WHO) grades of glioma rise [3]. Moreover, it is not possible to isolate TSs from GBM specimens in all cases, with reported isolation rates estimated at 43.8 % [3]. Notably, the ability to isolate TSs is a significant prognostic factor for overall survival in patients with primary GBM [15].

It is known that the weight of fresh specimens varies from one surgical procedure to the next. This raises the question of whether the weight of a specimen is a determining factor in the ability to isolate TSs from it. In some isolation protocols, it is suggested that a specimen weight of 200–500 mg is needed for isolation of TSs [11]. However, there is no established experimental relationship between the weight of fresh specimens and the isolation of TSs.

In this study, we assessed the predictive value of fresh specimen weight in determining the ability to isolate TSs, testing the hypothesis that the greater the weight of the specimen, the more effective the isolation of TSs. Thirty-five fresh specimens of WHO grade IV gliomas were divided into two groups, based on the ability to isolate TSs from them, and the relationship between specimen weight and TSs isolation was studied. We also evaluated the optimal cut-off weight of specimens for the isolation of TSs.

## Methods

### Patient population

Patients with WHO grade IV glioma treated at our institution between October 2014 and August 2015 were included in this study (Table 1). All patients were histologically diagnosed according to the 2007 WHO classification, and were graded by neuropathologists; the molecular properties of each surgical specimen have been reported [16]. O-6-methylguanine-DNA methyltransferase (MGMT) promotor methylation and isocitrate dehydrogenase (IDH)-1 mutation status were assessed by polymerase chain reaction (PCR) and immunohistochemistry (IHC). In cases where IHC results for IDH1 were negative, we tested for IDH1 mutations using the hot-spot technique. Epidermal growth factor receptor (EGFR) and loss of heterozygosity (LOH) at chromosomes 1p and 19q were evaluated by fluorescence in situ hybridization (FISH). P53 was identified by IHC.

**Table 1:** Demographic and clinical characteristics of patients with WHO grade IV glioma

Characteristics	TS culture positive (n = 18)	TS culture negative (n = 17)	P value*
Age (years)	62.6 ± 10.7	55.5 ± 12.9	0.088
Sex (M:F)	10:8	10:7	0.845
Pathological diagnosis			>0.999
GBM	14 (77.8 %)	15 (88.2 %)	
GBMO	2 (11.1 %)	1 (5.9 %)	
Gliosarcoma	2 (11.1 %)	1 (5.9 %)	
Type			>0.999
Primary	16 (88.9 %)	15 (88.2 %)	
Recurrent	2 (11.1 %)	2 (11.8 %)	
Molecular markers			
IDH1			
Wild type	18 (100 %)	17 (100 %)	
Mutation	0	0	
1p19q			>0.999
No deletion	16 (88.9 %)	15 (88.2 %)	
Codeletion	2 (11.1 %)	2 (11.8 %)	
MGMT promotor			0.358
Unmethylated	10 (55.6 %)	12 (70.6 %)	
Methylated	8 (44.4 %)	5 (29.4 %)	
P53 mutation			0.443
Wild type	3 (16.7 %)	5 (29.4 %)	
Positive by IHC	15 (84.3 %)	12 (70.6 %)	
EGFR mutation			0.193
Wild type	8 (44.4 %)	4 (23.5 %)	
Positive by FISH	10 (55.6 %)	13 (76.5 %)	

* By Independent two-sample *t* test for continuous variables and Chi square test (or Fisher’s exact test) for categorical variables

### From fresh specimen to single cell isolation

Fresh tumor specimens were obtained in the operating room from glioma patients undergoing surgery. Each specimen was place in a sterile centrifuge tube (SPL Life Sciences Co., Ltd, Korea) on ice, and was weighed on the same electronic precision balance (Sartorius^®^ TE4101-L, Sartorius Weighing Technology GmbH, Goettingen, Germany) within 1 h. Thereafter, specimens were processed using a previously reported mechanical dissociation method [1, 3, 17]. Briefly, surgical specimens were minced and dissociated with a scalpel in Dulbecco’s modified Eagle medium/nutrient mixture F-12 (DMEM/F-12; Mediatech, Manassas, VA, USA) and then passed through a series of 100-μm nylon mesh cell strainers (BD Falcon, Franklin Lakes, NJ, USA). Cell suspensions were washed twice in DMEM/F-12 and cultured in complete media (DMEM/F-12) containing 1xB27 supplements (Invitrogen, San Diego, CA, USA), 20 ng/ml of basic fibroblast growth factor (bFGF; Sigma, St. Louis, MO, USA), 20 ng/ml of epidermal growth factor (EGF; Sigma), and 50 U/ml penicillin/50 mg/ml streptomycin [1, 3, 17].

### Isolation of TSs

Isolated single cells were cultured as gliomaspheres in complete TS medium consisting of DMEM/F-12 containing 2 % 1× B27, 20 ng/ml of 0.02 % bFGF, 20 ng/ml of 0.02 % EGF, and 1 % antibiotic–antimycotic solution (100× ; Gibco, Invitrogen Korea, Seoul, Korea). The cells were cultured continuously through three to six passages, consistent with their status as progenitor/stem cells. Cell morphology was assessed by observing cultures with an inverted phase-contrast microscope (I × 71 Inverted Microscope; Olympus, Tokyo, Japan). The neural differentiation potential of gliomaspheres was subsequently tested, followed by an evaluation of their ability to induce tumorigenesis in vivo. The relationship between the isolation of TSs and the weight of surgical specimens was investigated, and the optimal cut-off weight for isolation of TSs was evaluated.

### Immunocytochemical staining

For investigation of surface and intracellular antigen expression profiles, TSs were transferred to cover slides, fixed with 2 % paraformaldehyde for 7 min, and then treated with a 3:1 ratio of methanol and acetic acid for 3 min. The cells were then washed and permeabilized by incubating with 0.1 % Triton X-100 for 10 min. After blocking with 1 % bovine serum albumin (BSA; Amresco, Solon, OH, USA) for 1 h, cells were incubated with primary antibodies for 2 h at room temperature. The following antibodies were used: rabbit anti-CD133 (1:250, ab19898; Abcam [Dawinbio Inc], Hanam, Korea), rabbit anti-nestin (1:250, ab5968; Abcam). Primary antibodies against CD133 and nestin were detected with goat anti-rabbit IgG conjugated with Alexa Fluor 555 (1:2000; Invitrogen), which is spectrally similar to Cy3. The cells were mounted with Vectashield H-1200 mounting media containing 4′6-diamidino-2-phenylindole (DAPI; Vector Laboratories, Burlingame, CA, USA) to counterstain nuclei. Phosphate-buffered saline (PBS; Dawinbio Inc, Hanam, Korea) was used for all washing steps, and antibody diluent reagent solution (Invitrogen) was used to dilute antibodies. As a negative control, only the secondary antibody was used. A fluorescence microscope (1 × 71; Olympus Korea, Seoul, Korea) and DP Controller software (Olympus Korea) were used for observing and photographing the cells.

### Immunohistochemical staining

Sections (3-mm thick) were deparaffinized in xylene and rehydrated through a graded alcohol series to distilled water. Antigen retrieval was performed by microwave irradiation, after which samples were incubated with the following primary antibodies: rabbit polyclonal anti-CD133 (1:200, ab19898; Abcam [Dawinbio Inc], Hanam, Korea), mouse monoclonal anti-nestin (10C2; CELL MARQUE, Rocklin, CA95677, USA), and mouse monoclonal anti-CD15 (1:50, M3631; Dako Korea LCC, Seoul). Specific binding was detected using biotinylated anti-mouse IgG, followed by peroxidase/alkaline phosphatase streptavidin, with 3,3′-diaminobenzidine and the combination of nitro blue tetrazolium chloride (NBT) and 5-bromo-4-chloro-3-indolyl phosphate (BCIP) as substrates.

### Neuro-glial differentiation

The multipotency of TSs was tested by examining neural lineage expression by immunocytochemical staining. Briefly, after being seeded onto chamber slides (Lab-Tek II; Nalge Nunc International, Rochester, NY, USA), cells were grown in neural differentiation media containing 10 % fetal bovine serum (FBS; Lonza) and 1 × B27 supplement (Invitrogen) for up to 14 days. Cells were then fixed with 2 % paraformaldehyde for 7 min at 4 °C, and permeabilized by incubating with 0.1 % Triton X-100 for 10 min. After blocking with 1 % BSA (Amresco) for 1 h, cells were immunostained with the following antibodies: rabbit anti-GFAP (1:200 dilution; Dako, Carpinteria, CA, USA), mouse anti-MBP (myelin basic protein, 1:200 dilution; Chemicon, Temecula, CA, USA), mouse anti-NeuN (1:100 dilution; Chemicon), and mouse anti-TUBB3 (Tuj1, 1:200 dilution; Chemicon). The primary antibodies were detected with Cy3-conjugated anti-mouse or anti-rabbit secondary antibodies (1:200 dilution; Jackson ImmunoResearch Laboratories, West Grove, PA, USA), as appropriate. Nuclei were counterstained with DAPI (Vector Laboratories). Slides were examined and photographed using a fluorescence microscope.

### Statistical analysis

The patients’ demographic characteristics and weight of each surgical specimens were compared using the independent two-sample *t* test for continuous variables and Chi square test (Fisher’s exact test) for categorical variables. Youden’s method in conjunction with receiver-operating characteristic (ROC) analysis was used to determine the optimal cut-off weight of fresh specimens for isolation of TSs to maximize sensitivity and specificity. All statistical analysis were performed using SAS version 9.2 software (SAS Institute Inc. Cary, NS, USA), MedCalc version 15.0 software (MedCalc Software, Ostend, Belgium) and SPSS version 18.0 KO software (SPSS Korea, Seoul, Korea), with P < 0.05 considered statistically significant.

## Results

### Patient population

A total of 35 fresh surgical specimens were collected from 20 males and 15 females, ranging in age from 33 to 77 years, during the period of October 2014 to August 2015. Pathological diagnoses included GBM (n = 29), GBM with an oligodendroglial component (n = 3), and gliosarcomas (n = 3). There were 31 primary and 4 recurrent types of WHO IV gliomas. Of these 35 specimens, 18 were categorized as positive and 17 as negative for TS isolation (Table 1). Molecular factors, including IDH-1 mutation, MGMT promotor methylation, EGFR, p53, 1p and 19q LOH were evaluated. There were no statistically significant differences in age (P = 0.088), sex (P = 0.845), pathological diagnosis (P > 0.999), type (P > 0.999), 1p 19q codeletion (P > 0.999), MGMT promotor methylation (P = 0.358), p53 (P = 0.443), or EGFR mutation status (P = 0.193) between TS-positive and TS-negative groups.

### Characterization of GBM TSs

**Fig. 1:**
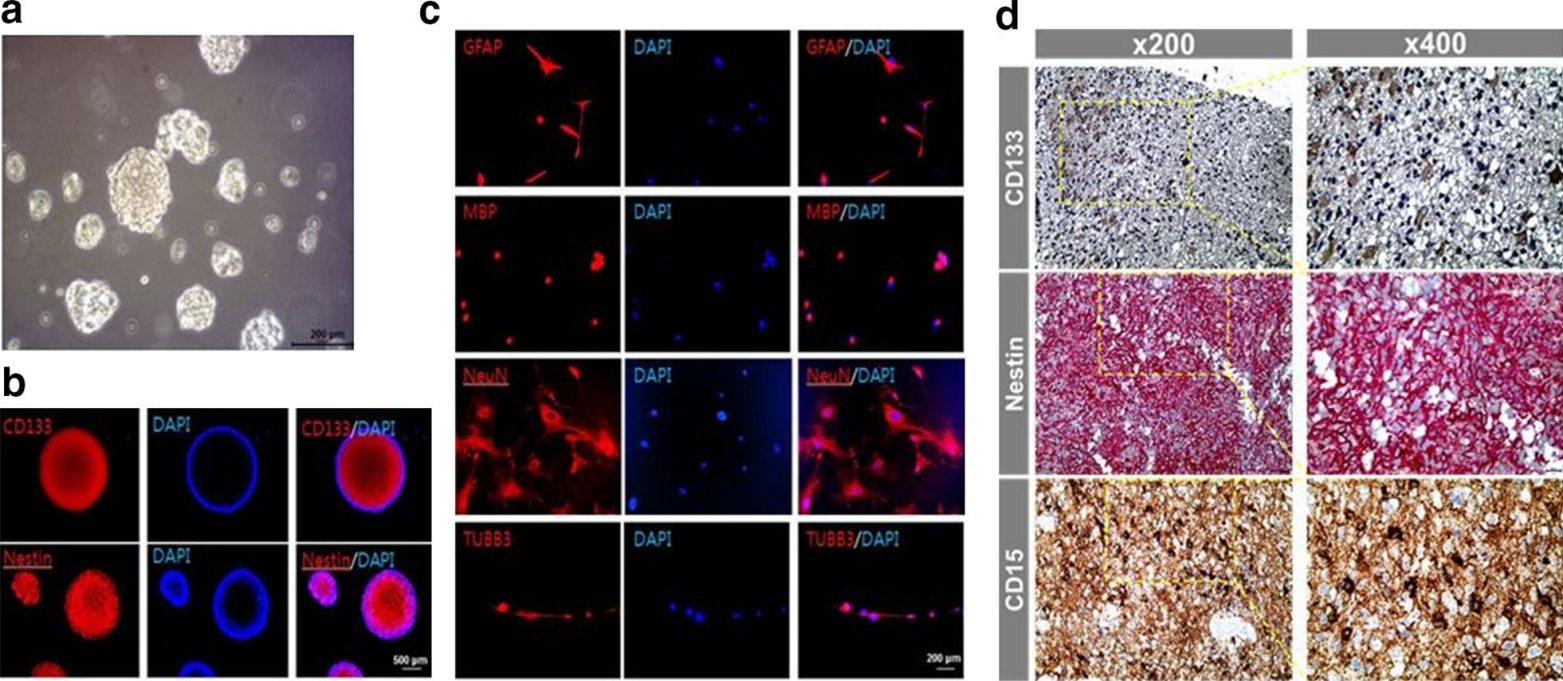
Characterization of a representative GBM TS (TS15-88). **a** Morphology of TSs shown by phase-contrast microscopy (×100 original magnification). **b** Immunocytochemical staining of TSs for CD133 and nestin; nuclei were counterstained with DAPI (×100 original magnification). **c** TSs grown in neural differential media were immunostained for GFAP, MBP, NeuN, and TUBB3 (×200 original magnification). **d** IHC staining for CD133, CD15 and nestin in the GBM specimen from which this TS sample (TS15-88) was isolated, showing that these markers were well expressed (×200, ×400 original magnification) in this GBM specimen

Cells isolated from tumor specimens yielded spheroids when cultured in TS complete media (Fig. 1a). An immunocytochemical analysis of a representative TS sample (TS15-88) identified cells expressing markers associated with stem cells and brain tumor stem cells, including CD133 and nestin (Fig. 1b). To assess the multilineage differentiation capacity of TSs, we cultured them in neuro-glial differentiation media, as described in “Methods” section, and analyzed them for the expression of the differentiation markers TUBB3 (immature neurons), GFAP (astrocytes), MBP (oligodendrocytes), and NeuN (mature neurons) by immunocytochemical staining (Fig. 1c). The GBM specimen from which this TS sample was derived was evaluated by IHC staining for CD133, CD15 and nestin (Fig. 1d), which showed that all of these markers were well expressed in this GBM specimen.

### Weights of fresh specimens

**Fig. 2:**
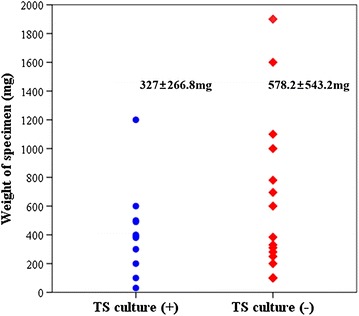
Weight distributions of fresh specimens in the two groups. There was no significant difference in the weight of surgical samples in the two groups (P = 0.245, Mann–Whitney test)

The average weights of fresh specimens were 327 ± 266.8 mg in the TS-positive group and 578.2 ± 543.2 mg in the TS-negative group (Fig. 2). Our original expectation was that the greater the weight of the sample, the more likely the isolation of TSs. Contrary to our expectations, the mean weight for the TS-negative group trended larger than that for the TS-positive group, although this difference did not reach statistical significance (P = 0.245). Thus, our data indicate no association of the weight of specimens with the ability to isolate TSs.

### The optimal weight of specimens for TS isolation

**Fig. 3:**
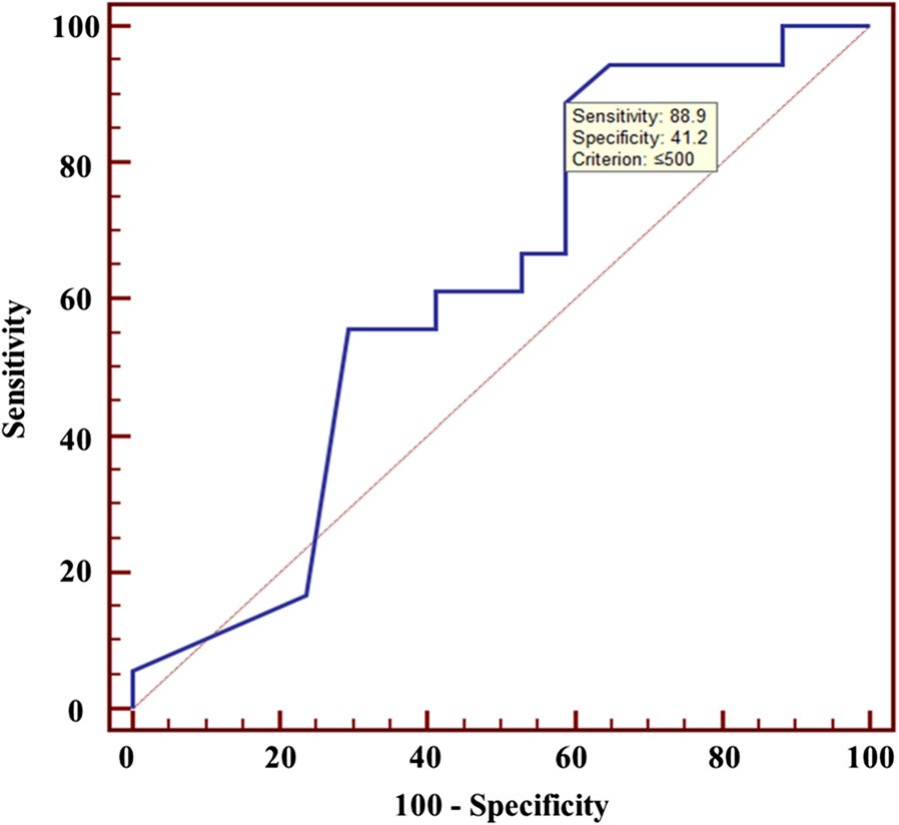
Receiver-operating characteristic curve for isolation of TSs. The optimal cut-off value to maximize the sensitivity and specificity of TS isolation from WHO IV gliomas was 500 mg

The optimal cut-off value for isolation of TSs was determined by measuring the area under the ROC curve of 0.618 (Fig. 3). This translated to an optimal fresh specimen weight of 500 mg for isolation of TSs, with a sensitivity of 88.9 % and specificity of 41.2 %. In contrast to our initial hypothesis that larger specimens would be associated with higher rates of TS isolation, we found that the sensitivity and specificity of TS isolation was actually higher in specimens weighing less than 500 mg than in those greater than 500 mg.

## Discussion

It has been assumed that TSs arise from a subpopulation of cells responsible for the initiation, maintenance, and recurrence of tumors [2, 18]. It is also commonly thought that TS-generating cells comprise some proportion of the tumor, and that a higher proportion of these TS-generating cells would be associated with a more aggressive tumor [3, 15, 19]. A previous study reported a significant increase in TS isolation rate in gliomas with increased WHO grades [3]. This suggested the related hypothesis that a larger specimen mass would tend to favor an increased TS isolation rate. Our test of this hypothesis, however, revealed no significant difference in specimen weights between TS-positive and -negative groups. We also found that the two groups were not different with respect to clinical characteristics, molecular factors, or pathological features that might influence TS isolation.

Siedel et al. [12] reported that most tumor samples with a size up to 0.5 cm^3^ are suitable for GBM TSs isolation. Some authors recommend 200- to 500-mg specimens for isolation of GBM TSs [11], and there have been some suggestions about the weight or volume of specimens for isolation of TSs from other kinds of cancers [5, 9, 20]. Most other TS-related studies have provided no description of the weight or volume of fresh specimens for TS isolation [19, 21–24]. Generally speaking, reports such as those referred to above have assumed that, all other things being equal, larger specimens are better than smaller specimens for TS isolation [5, 7, 9, 11, 12]. However, no studies have compared TS-negative and -positive groups to establish optimal weights or volumes for TS isolation.

It is difficult to isolate TSs, and the efficacy of the procedure is low (from 1 to 30 %) [23, 25, 26]. Most studies on malignant tumors of the brain and other organs have reported TS isolation efficiencies up to 40–60 % [2, 3, 9, 15, 27, 28], similar to the results of the results of our current study. However, some authors have described sequential modifications of isolation techniques that improved efficiency from 40 to 90 % in GBM patients [18]. In this latter case, improvements in TS isolation involved fixes to technical problems associated with each stage, such as early vulnerability of surgical specimens, mechanical disruption, and removal of red blood cells and necrotic material from fresh specimens. Although studies such as this highlight the contribution of technical problems to the isolation of TSs, the fact that the ability to isolate TSs is correlated with poorer clinical outcome in various types of cancers [3, 15, 29–33] indicates that success of TS isolation is not solely determined by technical issues.

Previous report proposed that primary GBM TSs isolation is a prognostic indicator of clinical outcome [15]. In addition, some authors described that TSs isolation is supposed to be a poor prognostic factor in other malignant gliomas [34]. In the current study, we evaluated the prognostic role of TSs in WHO grade IV gliomas. TS isolation from WHO grade IV gliomas were not significantly related to overall survival (data not shown), and there were no significant differences in clinical characteristics, molecular factors, or pathological features between TS-positive and TS-negative groups (Table 1). However, these results could be related to the short follow-up period (10 month), suggesting that long-term follow-up of this cohort is needed to assess the prognostic role of TS isolation in patients with WHO grade IV gliomas.

There are some concerns that surgical procedures used to collect fresh specimens can affect TS isolation. Some authors have reported that surgical procedures can help TSs exit from quiescence, inducing the formation of more TSs for analysis consequently [19]. On the other hand, surgically resected tissue is vulnerable to rapid ischemic and degenerative alterations [7, 19]. Thus, optimal conditions, including rapid transfer of specimens following surgical resection, removal of red blood cells and necrotic material and an appropriate environment for growth are required for the isolation of TSs from fresh surgical specimens [5, 7, 9, 11, 12, 18, 19, 23, 35]. Our laboratory has an ongoing effort to develop optimal conditions for the isolation of TS [2, 3, 10, 17].

RNA sequencing or gene expression arrays could be helpful in validating new TS markers and targets. However, the genome analyses necessary to establish transcription factors that might influence to TS isolation have not been performed. In this context, it has been shown that functional inhibition of microRNA-138 (miR-138) in malignant glioma prevents TS formation in vitro and impedes tumorigenesis in vivo [36]. In our study, we sought to determine whether the ability to isolate TSs differed depending on the weight of the specimen. However, further studies regarding transcription factors necessary for TS isolation are needed.

In our study, the specimen weight was 500 mg for the isolation of TSs to maximize sensitivity and specificity. This result was a little similar to the value reported by other authors [11]. Ideally, specimens would be subdivided into various sizes and then cultured, or more than one specimen would be procured from a single patient. However, in actual practice, the operating room setting imposes limitations on specimen collection. We tried to collect specimens with a minimum of red blood cells and necrotic material, but this made it difficult to gather specimens of different sizes from each patient. Therefore, we emphasized the absence of significant differences between demographic and clinical characteristics of two groups, and used the ROC curve for statistical processing. An analysis of our data yielded an area under the ROC curve of 0.618, which demonstrated poor discriminatory ability for the isolation of TSs [37, 38]. However, 500 mg of fresh specimen proved to be optimal in our study, and our summary data showed no significant difference in weight between the TS-positive and TS-negative groups, clearly ruling out size/weight of sample as a determining factor in successful TS isolation. Our study suggests some methods for improving the efficiency of TS isolation in addition to stage-specific technical modifications. Future studies using a larger numbers of cases are warranted to further address the relationship between the amount of fresh specimen and TS isolation rate.

## Conclusion

Our initial hypothesis that a larger amount of specimen would translate to a higher rate of TS isolation proved to be incorrect. Instead, we found no relationship between the weight of specimen and TS isolation rate. Moreover, our data suggested that 500 mg of fresh specimen was optimal for isolation of TS with maximal sensitivity and specificity. The results of this study could provide useful technical information for cellular immortalization of patients with WHO grade IV gliomas.

## Abbreviations

TS: tumorsphere
WHO: World Health Organization
GBM: glioblastoma
MGMT: O-6-methylguanine-DNA methyltransferase
IDH: isocitrate dehydrogenase
PCR: polymerase chain reaction
IHC: immunohistochemistry
EGFR: epidermal growth factor
LOH: loss of heterozygosity
FISH: fluorescence in situ hybridization
ROC: receiver-operating characteristic
